# Does the pancreatic volume reduction rate using serial computed tomographic volumetry predict new onset diabetes after pancreaticoduodenectomy?

**DOI:** 10.1097/MD.0000000000006491

**Published:** 2017-03-31

**Authors:** Sung Pil Yun, Hyung-Il Seo, Suk Kim, Dong Uk Kim, Dong Hoon Baek

**Affiliations:** a Department of Surgery; b Department of Radiology; c Department of Internal Medicine, Biomedical Research Institute, Pusan National University Hospital, Busan, Korea.

**Keywords:** CT volumetry, new onset DM, pancreatic volume reduction rate, pancreaticoduodenectomy

## Abstract

Volume reduction of the pancreatic tissues following a pancreatectomy can lead to the deterioration of glucose homeostasis. This is defined as pancreatogenic diabetes mellitus (DM). The objective of this study was to investigate the occurrence of new-onset DM (NODM) and evaluate the risk factors, including the pancreas volume reduction rate in patients undergoing pancreaticoduodenectomy (PD).

Sixty-six patients without preoperative DM underwent PD for periampullary tumors between August 2007 and December 2012 and were included in this analysis. These patients underwent follow-up tests and abdominal computed tomography (CT) scan 7 days, 6 months, 12 months, 24 months, and 36 months after the operation. The pancreas volume reduction rate was calculated by CT volumetry. The patients were divided into 2 groups according to the postoperative development of DM.

After PD, newly diagnosed DM occurred in 16 patients (24.2%). The incidence of DM was highest among patients with carcinomas with an advanced T stage. The pancreatic volume reduction rate after 6 and 12 months in the NODM group was significantly higher than the normal glucose group in the univariate analysis. In the multivariate analysis, the pancreatic volume reduction rate 6 months after PD was the only significant predictive factor for the development of NODM (*P* = 0.002).

This study suggests that the pancreatic volume reduction rate 6 months after PD was the only significant predictive factor for the development of NODM. CT volumetry of the pancreas may be useful as a predictor of NODM after PD.

## 1 Introduction

Pancreatic resection could be a risk factor for new onset diabetes mellitus (DM) due to the deterioration of glucose metabolism caused by volume reduction of endocrine and exocrine pancreatic tissues.^[1]^ New onset DM after pancreatectomy has been defined as pancreatogenic diabetes, classified as type 3c by the American Diabetes Association.^[2,3]^ Among the 8% to 9% of the general diabetes population with type 3c diabetes in Western countries, 2% to 3% are caused by pancreatic resection.^[3–5]^ In a previous study, impaired glucose tolerance developed in approximately 25% of healthy adult patients who underwent pancreatectomy. Recent development of better diagnostic modalities and a better understanding of pancreatic disease has increased the frequency of pancreatectomies in benign or premalignant pancreatic disease. Distal pancreatectomy (DP) has been successful for various pancreatic diseases, leading to long-term survival. Because of the success in mortality rates, several studies have now focused on the morbidity of the surgery, namely, new onset DM. Several research studies have reported that new onset DM developed in 4.8% to 38% of patients after DP.^[3,6–8]^ And, after PD, the incidence of new onset DM was reported between 0% and 50%.^[3,6–16]^ However, with pancreaticoduodenectomy (PD), lower survival rates and poor prognosis have been reported due to the high rate of pancreatic head cancer compared to other malignant tumors in the periampullary lesion. For this reason, new onset DM after PD has received very little attention. However, there is a concern that underestimation of the incidence and clinical importance for new onset DM might cause decreased quality of life by delaying proper medical therapy. Additionally, it is important to quantify the risk of new onset DM because other malignancies and premalignant lesions for which PD is performed, such as ampulla of Vater cancer or intraductal papillary mucinous neoplasm, have expectation for long-term survival. Although there is more research on DP than for PD in this matter, reliable risk factors for new onset DM have not been determined due to limitations of the studies. Recently, some studies attempted to predict new onset DM by assessing the degree of pancreatic volume reduction using CT volumetry. The objective of this study was to investigate the frequency and risk factors of new onset DM in patients who have undergone PD and had long-term survival. In addition, we performed pancreatic volume analysis to examine the relationship between the serial reduction in pancreatic volume and the development of new onset DM, perhaps revealing the importance of evaluating glucose metabolism during follow-up.

## 2 Methods

### 2.1 Patients

We analyzed 113 patients who underwent PD for periampullary tumors at Pusan National University Hospital between August 2007 and December 2012. We excluded patients with preoperative DM or who had chronic pancreatitis or those who were lost to follow-up or who survived less than 3 years. Sixty-six patients met the criteria and were enrolled in the study. The patients were divided into 2 groups according to the postoperative development of DM: non-DM group (n = 50) and postoperative DM group (n = 16). These patients were retrospectively reviewed for age, sex, body mass index (BMI), postoperative pancreatic fistula (POPF), postoperative adjuvant therapy, pathologic characteristics, presence of cardiovascular comorbidity and operative factors (operation type, transfusion) based on the medical records. These patients also had the volume of their pancreas measured by computed tomography (CT) volumetry before surgery, and then the remnant was measured on postoperative day 7 and months 6, 12, 24 and 36. This retrospective study was approved by the institutional review board at Pusan National University Hospital Clinical Trial Center (IRB No. E2016047), and written informed consent was obtained from all participants.

### 2.2 CT volumetry

**Figure 1:**
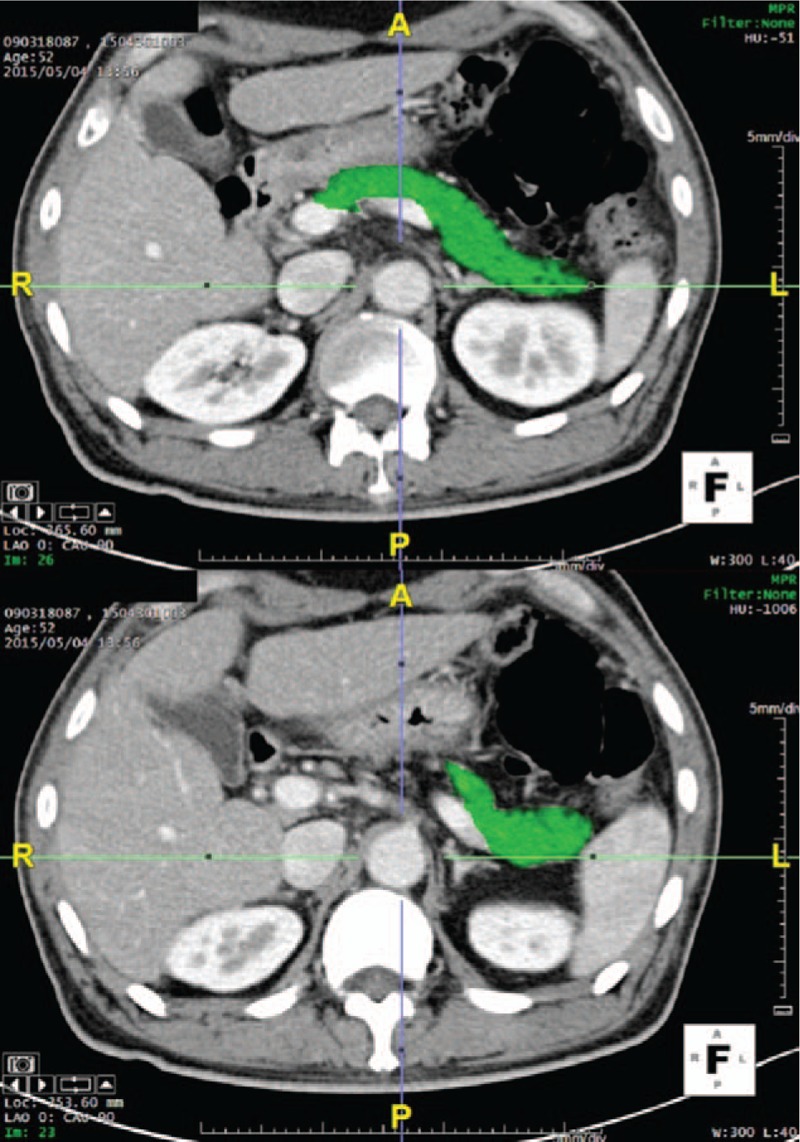
CT images of pancreatic phase were transported from PACS (Picture Archiving and Communication System) to the TeraRecon (San Mateo, CA) server. We drew the border of the pancreas by using the free-ROI manual method in the axial image.

Dynamic CT was performed using a 64-MDCT scanner (Discovery CT 750 HD; GE Healthcare, Milwaukee, WI). Images of the arterial, portal venous, and delayed phases were obtained using a helical scanning technique. The parameter of the CT scanners was 120 kVp and 0.625 mm collimation with a pitch of 0.984. Automatic tube current modulation was applied with a setting from 180 to 350 mA. One hundred twenty milliliters of Omnipaque 300 (GE Healthcare, Princeton, NJ) was injected at a rate of 3 mL/s. Arterial phase, pancreatic phase and delayed phase imaging were acquired at 35, 70, and 125 seconds, respectively, after the contrast material was injected. The axial images were reconstructed at a 3-mm section thickness. Three-dimensional (3D) images were reconstructed by TeraRecon Aquarius Workstation v4.4 (TeraRecon, Inc., San Mateo, CA) using an LD2 algorithm (window 300, level 40) with axial images (Fig. 1). The volume of the pancreatic parenchyma was easily calculated within the TeraRecon program at all time points. In addition to describing the resection volume (initial volume–postoperative 7 days volume), we measured the pancreas volume reduction rate (the current pancreas volume/volume of the preoperative whole pancreas) at 7 days after surgery and then at 6, 12, 24, and 36 months.

### 2.3 Definition of DM

Preoperative DM was defined as the current use of oral hypoglycemic agents or insulin. New onset DM was defined by one or more of the following: initiation of hypoglycemic agent (oral agent or insulin), fasting plasma glucose of ≥126 mg/dL after fasting for ≥8 hours, or a 2-hour postprandial glucose level more than 200 mg/dL at follow-up.^[2,10]^

### 2.4 Statistical analysis

Data distribution was verified by the Shapiro–Wilk test. Categorical variables between 2 groups were compared using the Chi-square or Fisher exact test. The continuous variables were compared using Students *t* test or the Mann–Whitney *U* test, where appropriate. A logistic regression model was used for univariate and multivariate analyses. The cutoff value of the pancreas volume reduction rate for predicting new onset DM was determined by a receiver operating characteristic (ROC) curve analyses. *P* values less than 0.05 were considered statistically significant. All statistical analyses were performed using the SPSS version 23.0 for Windows (SPSS, Inc., Chicago, IL).

## 3 Results

A total of 66 patients were included in the analysis. Demographics and clinical characteristics of the patients are summarized in Table 1. The mean age was 62 years, and 43 of the patients were males (63.6%). The mean BMI was 23.28 ± 3.26 kg/m^2^. Pylorus preserving pancreaticoduodenectomy (PPPD) was performed in 44 patients (66.7%), and 22 patients (33.3%) underwent PD. The tumor locations included the pancreatic head (n = 12, 18.2%), the bile duct (n = 26, 39.4%), ampulla of Vater (n = 27, 40.7%), and the duodenum (n = 1, 1.7%). Postoperative pathologic results revealed malignancy in 60 patients, while the pathology was benign in the other.^[6]^ The benign diseases included intraductal papillary mucinous neoplasm, solid and papillary epithelial neoplasm, and paraduodenal pancreatitis. The median duration of follow-up after surgery was 40 months. Postoperative new-onset DM (NODM) had developed in 16 (24.2%) of the 66 patients at an average of 14.8 months after surgery.

**Table 1:**
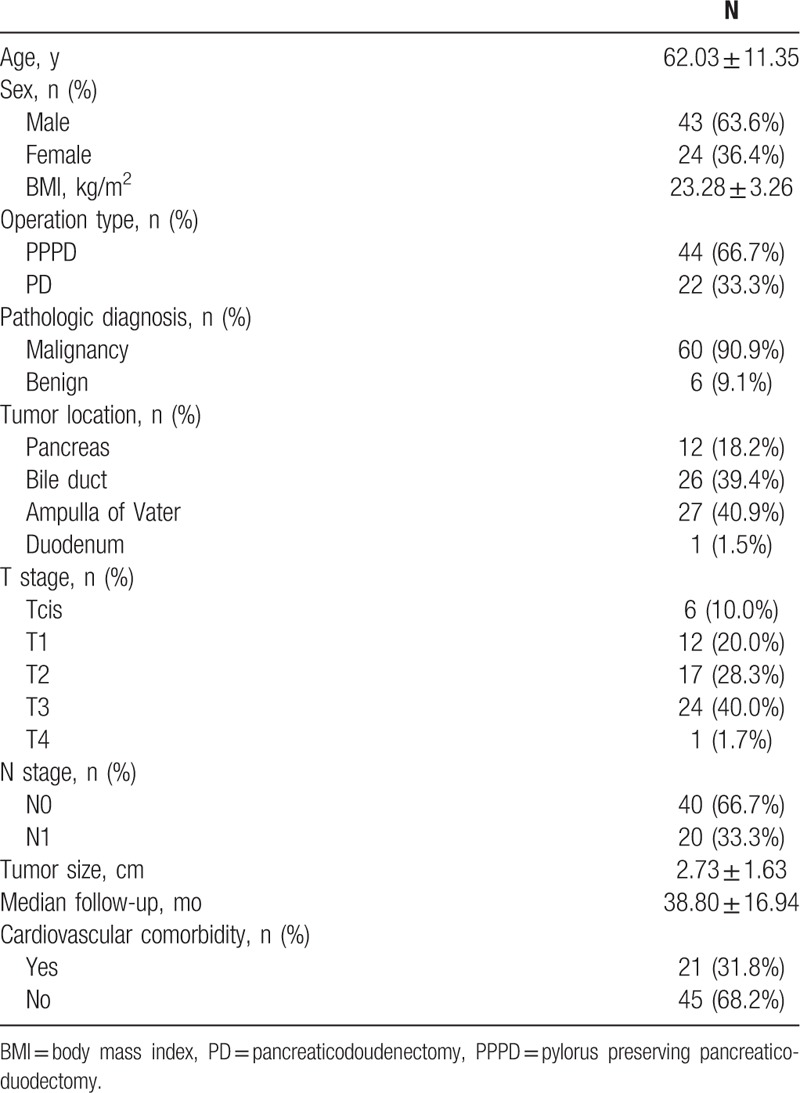
Demographics and clinical characteristics of patients (n = 66).

Comparison of clinicopathologic factors between 2 groups are described in Table 2. Age, sex, BMI, BMI above 25 kg/m^2^, operation type, transfusion, pancreatic fistula, adjuvant treatment, N stage, recurrence, tumor size, cardiovascular comorbidity, cholesterol level, anesthesia time, perineural invasion, and lymphvascular invasion were not significantly different between the normal and new onset DM groups. An advanced T stage was identified as a risk factor for new onset DM after PD in univariate analysis (*P* = 0.035).

**Table 2:**
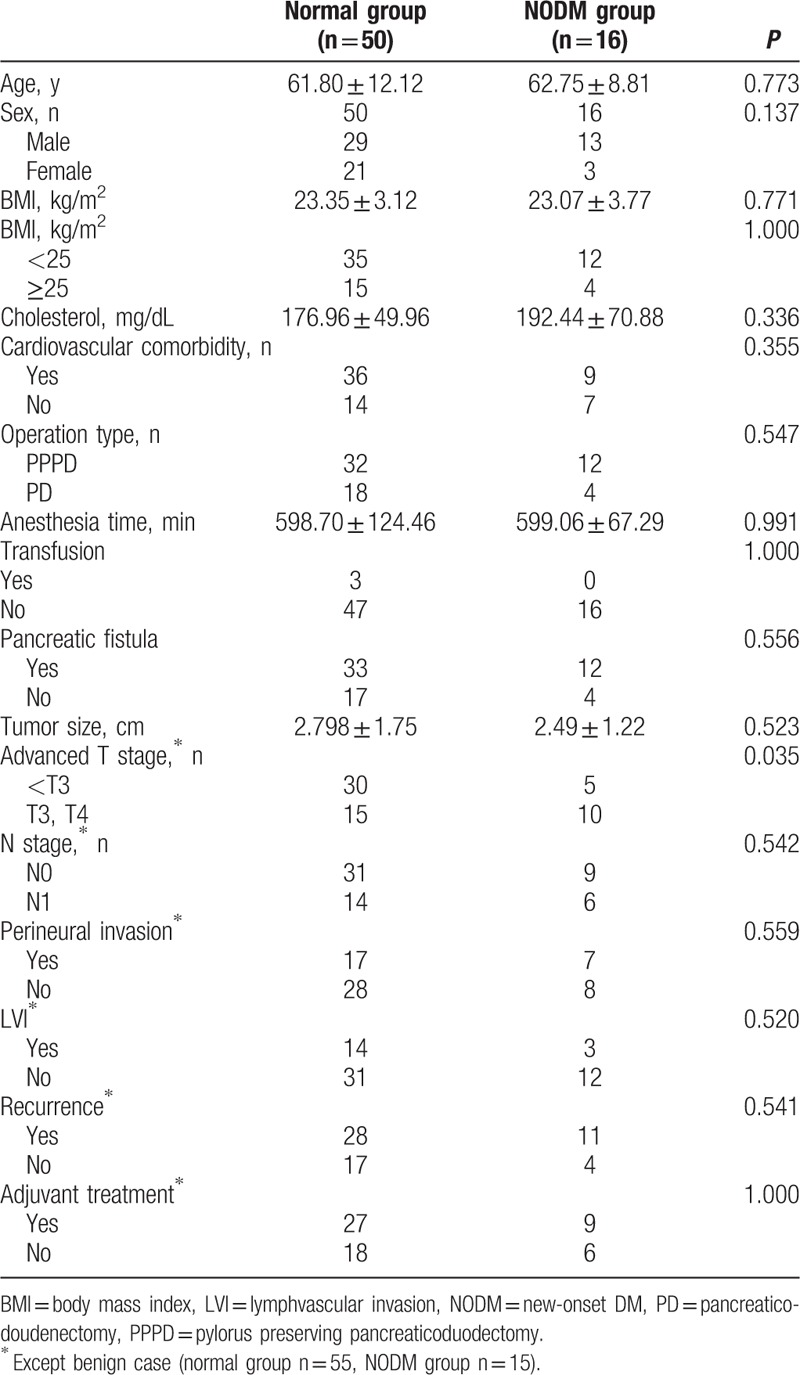
Comparison of clinicopathologic factors between the normal group and postoperative diabetes mellitus group.

**Figure 2:**
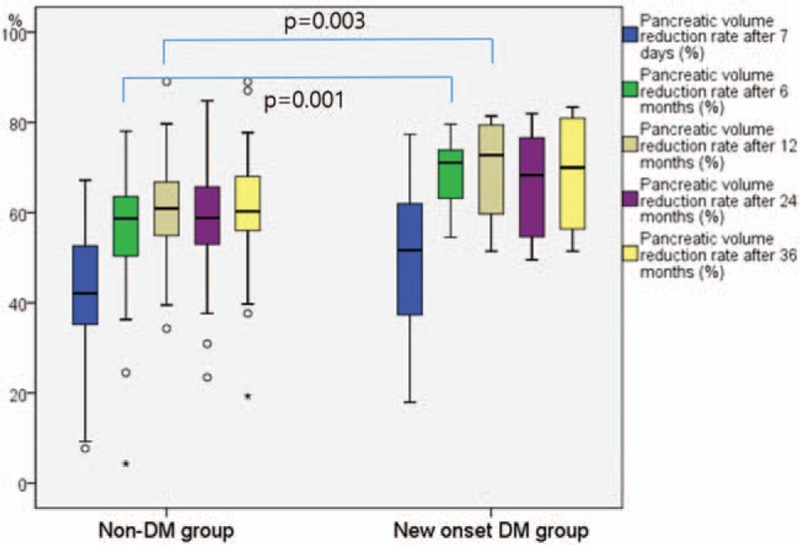
Boxplots showing pancreatic volume reduction rate all time points after pancreaticoduodenectomy.

The mean pancreatic resection volume on CT volumetry was 36.60 ± 18.13 mL. The mean pancreatic resection volume did not differ between the normal glucose group and the new onset DM group (34.51 ± 17.65 mL vs 43.14 ± 18.59 mL, respectively, *P* = 0.097). The pancreatic volume reduction rate after 6 and 12 months in new onset DM group was significantly higher than in the normal glucose group (56.75 ± 13.40% vs 69.25 ± 7.19%, at 6 months, respectively, *P* = 0.001 and 60.75 ± 10.05% vs 70.35 ± 11.02%, at 12 months, respectively, *P* = 0.003) (Table 3) (Fig. 2). There were no significant differences between groups in the pancreatic volume reduction rate at 7 days after PD, 24 months and 36 months. In the multivariate analysis, the pancreatic volume reduction rate 6 months after PD was the only significant predictive factor for the development of new onset DM (*P* = 0.002) (Table 4). The ROC curve analysis demonstrated the best fit when the cutoff value was a pancreatic volume reduction rate at 6 months of 64% (*P* = 0.000) (AUC = 0.809, sensitivity = 0.750, specificity = 0.776).

**Table 3:**
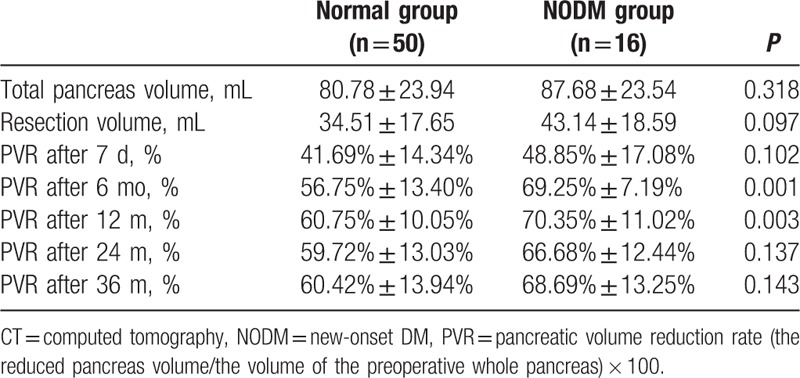
CT volumetry in pancreaticoduodenectomy patients.

**Table 4:**
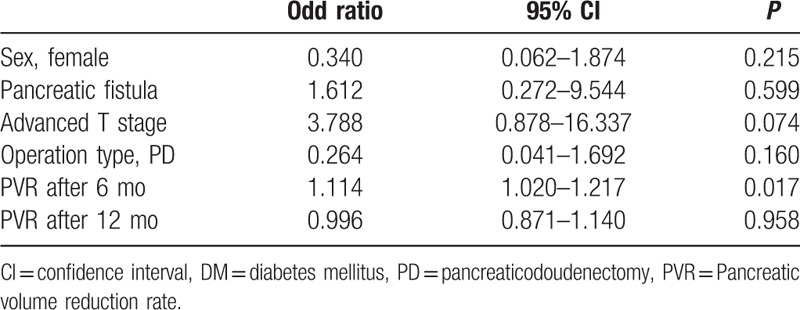
A multivariate logistic regression analysis of predictive factors for new onset DM.

## 4 Discussion

This study investigated the development of new onset DM after PD in patients with benign or malignant pancreatic tumors. It was designed to analyze whether there was a relation between new onset DM and the pancreatic volume after PD. Additionally, other clinical and pathologic factors were analyzed to determine if they were risk factors for new onset DM after PD. There have been various results from studies on the occurrence of new onset DM after PD, with the incidence reported between 0% and 50%.^[3,6–15]^ In this study, we report that PD led to glucose impairment, and postoperative NODM developed in 24.2% of nondiabetic patients who underwent PD. This result is consistent with the previous literature on the incidence of new onset DM after PD.^[3,6–16]^

Previously, several observational studies have demonstrated a risk of new onset DM after pancreatic surgery. Risk factors reported to be associated with new onset diabetes include BMI, resection volume, age and presence of pancreatitis.^[1,7,12]^ However, other studies have been unable to demonstrate statistically significance in these risk factors.^[10,17]^ King et al^[17]^ showed no significant risk factors for the development of new onset DM in a retrospective analysis of 125 patients after DP. You et al.^[10]^, in a study including 55 patients who underwent PD, described no significant clinicopathologic risk factors for new onset DM. In a univariate analysis, the present study indicated that advanced T stage was a risk factor of new onset DM after PD compared with the normal glucose group.

There has been very little research conducted examining the relationship between pancreatic volume status after pancreatectomy, especially PD and new onset DM. Thus, the association between the volume status after pancreatectomy and new onset DM is unclear and controversial. The exact volume of pancreas or resection volume cannot readily be determined during surgery. Therefore, the volume of the remnant pancreas after PD can be indirectly measured by CT volumetry.^[10]^ CT volumetry is a reliable method for estimating the endocrine function of the remnant pancreas.^[3,15]^ Some studies have analyzed the relationship between volume status (resection volume or remnant volume) and new onset DM and have shown that the pancreatic volume status after surgery did not significantly predict new onset DM.^[10,15]^

In the present study, our analysis also demonstrated that the resection volume during PD was not associated with new onset DM. However, our multivariate logistic regression analysis indicated that the pancreatic volume reduction rate 6 months after PD independently predicted the risk of new onset DM. This result indicated that atrophy of the remnant pancreas might be a more significant risk factor for new onset DM than the initial resection volume of the pancreas. The pancreatic volume reduction rate at 6 and 12 months after PD in the new onset DM group was significantly higher than the normal glucose group in the univariate analysis. This result proposes that the remnant pancreas volume decreased gradually until 12 months after PD. Therefore, postoperative atrophy of the remaining pancreas may significantly increase the risks of new onset DM. Although the mechanism of postoperative atrophy is not well clarified, a suggested mechanism involved the loss of the gastrointestinal hormones, such as cholecystokinin and gastrin, due to resection of the duodenum and distal stomach.^[6]^

In the 16 patients who developed new onset DM, the mean interval between PD and new onset DM was 14 months. This finding may be associated with our results, in which the pancreatic volume reduction at 6 and 12 months after PD is related to new onset DM. Similar results have been reported by Kwon et al.^[15]^ They described that the mean time interval between pancreatic resection and onset of DM was 16.8 months and suggested that careful analysis of glucose metabolism parameters is necessary, especially at approximately 16 months after pancreatic resection.

The difference between our research and previous studies is that by serial measurement of the remnant pancreatic volume, we drew the conclusion that the volume decrease at a particular point was associated with new onset DM. Based on our results and previous studies, the regular assessment of DM and follow-up should be considered within 18 months. The degree of atrophy appears to become stable within 18 months after surgery, thus, the rate of new onset DM is likely to decrease after that time point. In addition, this study indicates that if the pancreatic volume reduction rate is greater than 64% by 6 months, new onset DM can be predicted with a sensitivity of 75% and a specificity of 78% (AUC = 0.809). Therefore, if more than a 64% reduction is observed on CT volumetry, an evaluation for new onset DM should be performed.

The limitations of this study include its retrospective nature and the limited number of patients. We did not address why some patients developed more losses of pancreatic volume due to loss of the gastrointestinal hormones. And, the relationship between stricture of pancreaticojejunostomy and NODM in patients developing DM is not well clarified in this study. Additional large studies will be needed to clarify the predictive risk factors for new onset DM and the effect of pancreatic atrophy on new onset DM after PD.

## 5 Conclusion

In conclusion, after PD, postoperative NODM had developed in 24.2% of the nondiabetic patients. The pancreatic volume reduction rate after 6 months after PD was the only significant predictive risk factor for the development of new onset DM. The remnant pancreas volume decreased gradually until 12 months after PD. Therefore, postoperative atrophy of the remaining pancreas may significantly increase the risks of new onset DM. In addition, patients who undergo PD should be evaluated for new onset DM in order to improve their quality of life after surgery. Pancreatic volumetry may be a useful predictor for new onset DM during follow up.
